# Effect of *Helicobacter pylori*-eradication therapy on hepatic steatosis in patients with non-alcoholic fatty liver disease: a randomized–controlled pilot study

**DOI:** 10.1093/gastro/goz058

**Published:** 2019-11-25

**Authors:** Vikas Maharshi, Pooja Gupta, Vijay L Kumar, Ashish Datt Upadhyay, Prasenjit Das, Rajni Yadav, Baibaswata Nayak, Ramesh Kumar, Shalimar

**Affiliations:** 1 Department of Pharmacology, All India Institute of Medical Sciences, New Delhi, India; 2 Department of Biostatistics, All India Institute of Medical Sciences, New Delhi, India; 3 Department of Pathology, All India Institute of Medical Sciences, New Delhi, India; 4 Department of Gastroenterology, All India Institute of Medical Sciences, New Delhi, India

**Keywords:** non-alcoholic fatty liver disease, *H. pylori* infection, *H. pylori*-eradication therapy, controlled attenuation parameter, insulin resistance

## Abstract

**Background:**

*Helicobacter pylori* infection has been associated with insulin resistance and non-alcoholic fatty liver disease (NAFLD). This study was done to evaluate the effect of *H. pylori*-eradication therapy (HPET) in patients with NAFLD compared to standard management therapy (SMT).

**Methods:**

Eighty NAFLD patients with *H. pylori* co-infection were randomized into SMT (diet and exercise, *n *=* *36) and HPET (SMT plus amoxicillin, clarithromycin, and pantoprazole, *n *=* *44) groups. The controlled attenuation parameter (CAP), anthropometric parameters, liver enzymes, lipid profile, and glycemic parameters including homeostasis model assessment-insulin resistance (HOMA-IR) were measured and compared between two groups at the baseline and 24 weeks.

**Results:**

Sixty-four participants (SMT group [*n *=* *28] and HPET group [*n *=* *36]) were included in a modified intention-to-treat analysis. Both the SMT group and the HPET group had a significant reduction in CAP scores at 24 weeks (*P *=* *0.002 and *P *<* *0.001, respectively), but the change between the groups was insignificant (*P *=* *0.213). Successful eradication of *H. pylori* occurred in 68% of the HPET group and led to greater improvement in HOMA-IR at 24 weeks compared to SMT or non-responder patients (*P *=* *0.007). The liver enzymes reduced significantly at 24 weeks in both groups, but the changes between the groups were similar. The lipid parameters remained unchanged within the groups and between the groups at 24 weeks. A significant increase in the levels of reduced glutathione was noted in the HPET group, but the change between the two groups was not statistically different.

**Conclusions:**

HPET was found to be comparable to SMT alone in reducing hepatic steatosis and liver enzymes at 24 weeks in NAFLD patients. However, successful eradication of *H. pylori* led to greater improvement in HOMA-IR (Trial registration CTRI/2017/05/008608).

## Introduction

Non-alcoholic fatty liver disease (NAFLD) is characterized by ≥5% of hepatic steatosis that occurs due to causes other than significant alcohol consumption, medications, viral etiology, and autoimmune etiology [1]. NAFLD may progress to non-alcoholic steatohepatitis (NASH), cirrhosis, and hepatocellular carcinoma [1]. The current estimates suggest that 25% of the global population has NAFLD and, in India, its estimated prevalence ranges between 9% and 32% [1–3].

Insulin resistance (IR) is one of the most important factors for the development of hepatic steatosis [3]. Recent data suggest a possible role of *Helicobacter* *pylori* in the pathogenesis of NAFLD [4]. *Helicobacter* *pylori* infection has been associated with increased levels of pro-inflammatory cytokines and IR. A study from India has revealed a higher prevalence of *H. pylori* infection in diabetes as compared to controls [5]. Evidence suggests that *H. pylori* is involved in the regulation of gastric hormones leptin and ghrelin that affect insulin sensitivity and adiposity [6]. Though *H. pylori* is an important pathogen that should be eradicated whenever possible, the practice of detection and eradication of *H. pylori* in patients with NAFLD is not routinely followed in India. Moreover, the effect of *H. pylori* eradication on NAFLD per se is not clear. Currently, weight reduction remains the only option available as standard of care for the treatment of NAFLD. In a recent study among NAFLD patients with no hepatic fibrosis at baseline, the rate of progression of fibrosis was found to be related to the extent of hepatic steatosis [7]. Because *H. pylori* has an impact on IR and metabolic syndrome, this study was conducted to evaluate the effect of *H. pylori*-eradication therapy (HPET) in patients with NAFLD compared to standard management therapy (SMT) without *H. pylori* eradication.

## Patients and methods

This open-label, parallel group, randomized–controlled pilot study was carried out in a tertiary-care center in India between June 2017 and December 2018. The study was approved by the Institute Ethics Committee (IEC-236/05.05.2017, RP-25/2017) and was registered on the CTRI website (CTRI/2017/05/008608). The primary objective was to evaluate the effect of HPET on hepatic steatosis measured by controlled attenuation parameter (CAP) in NAFLD patients with *H. pylori* co-infection, at 24 weeks.

### Study subjects

All suspected NAFLD patients attending the Gastroenterology outpatient clinic were screened for eligibility. NAFLD patients of either gender, age ≥18 years and <65 years having *H. pylori* infection, and willing to give informed consent were included. Patients with endoscopy proven gastric/duodenal ulcer(s), simultaneous bacterial/viral/fungal infections requiring pharmacotherapy, history of intake of any anti-*H. pylori* pharmacotherapy within the past 1 year, history of allergy to any of the *H*. *pylori* triple-drug regimens (amoxicillin, clarithromycin, and pantoprazole) were excluded. Patients with alcohol consumption of >21 drinks/week for men and >14 drinks/week in women; coexisting hepatitis B or hepatitis C virus or human immunodeficiency virus infection; presence of other etiologies of liver disease-autoimmune hepatitis or Wilsons disease; previous liver surgery or liver transplantation; consumption of drugs known to cause fatty liver such as steroids, estrogens, methotrexate, tamoxifen, and amiodarone; unreliable FibroScan readings defined as interquartile range/median values of liver stiffness measurement (LSM) >0.3 were excluded. Patients with co-morbid conditions like diabetes mellitus, chronic kidney disease, liver cirrhosis, amyloidosis, and congestive heart failure were also excluded.

Ultrasound was used as the screening modality for the diagnosis of NAFLD. *Helicobacter* *pylori* infection was confirmed with histopathological examination of gastric antral biopsy and rapid urease test. Eligible NAFLD patients with *H. pylori* infection were randomized into either the SMT group or the HPET group by mixed block randomization. A random list was generated by the statistician using nQuery advisor version 7.1 software. Allocation of participants was concealed through a sealed opaque envelope method. The trial was conducted in accordance with the Declaration of Helsinki and Good Clinical Practice guidelines.

### Treatment details

Participants in the SMT group received diet and exercise advice. To maintain the uniformity in diet and exercise prescription, all the participants were explained to and were given a diet chart (provided by the hospital nutritionist) to provide energy equivalent to approximately 25 kcal/kg ideal body weight per day and an exercise chart to achieve metabolic equivalents of task (METs) of ∼1,000 per week.

Participants in the HPET group, in addition to SMT management, received a triple-drug regimen for *H. pylori* eradication—a Pantocid HP kit (procured from Sun Pharma Laboratories Limited). The Pantocid HP kit consists of two tablets each of amoxicillin 750 mg, clarithromycin 500 mg, and pantoprazole 40 mg. The pharmaceutical sponsor for the drugs had no involvement in protocol development, patient recruitment, and analysis of the data. Participants were instructed to take the medicines twice daily, 1 hour before meals for a duration of 14 days.

### Follow-up and outcomes

The primary outcome was the difference in the hepatic steatosis assessed by CAP between the two groups at 24 weeks. Secondary outcomes were changes in the IR, plasma lipids, liver enzymes, markers of inflammation (tumor necrosis factor-α [TNF-α]), oxidative stress (reduced glutathione [GSH]) and hepatic IR (adiponectin), and anthropometric parameters at 24 weeks with respect to their baseline values.

Participants were called for follow-up at the end of 6, 12, and 24 weeks following enrollment. Compliance to HPET was evaluated by drug history and by the pill-count method. Participants who completed the prescription as advised for 14 days were considered to be compliant. Any participant deviating from the said schedule was considered to be non-compliant. Compliance to dietary advice was measured by taking a dietary history. Participants achieving an average of at least 500 METs per week were considered to be compliant to exercise advice. Any adverse event experienced by the patients was recorded and appropriate investigation(s) were performed. A ^14^C urea breath test was performed at 6, 12, and 24 weeks through the Heliprobe^®^ system (KibionAB, Uppsala, Sweden) to assess the *H. pylori*-eradication response in the HPET group.

Anthropometric parameters, CAP-score assessment, liver-function tests, lipid profile, fasting plasma glucose (FPG), fasting insulin (FIns), and Homeostatic Model Assessment-Insulin Resistance Index (HOMA-IR; [FPG × Fins]/405, where FPG is in mg/dL and FIns is in μU/mL] [8, 9] were repeated at 24 weeks. Body composition and levels of serum TNF-α, GSH, and adiponectin were measured at baseline and at the 24-week follow-up visit. Serum TNF-α, GSH, and adiponectin were measured using commercially available ELISA kits (Elabscience^®^) as per the manufacturer’s protocol.

LSM and CAP measurements were performed using a FibroScan touch 502 (Echosens, Paris, France). A single operator performed all measurements. The examination was performed on the right lobe of the liver through the intercostal space. An M-probe was used for patients with a body mass index (BMI) <30 kg/m^2^ and XL-probe for patients with BMI ≥30 kg/m^2^. Ten successful acquisitions were performed for all patients as per the manufacturer’s instructions [10, 11].

### Statistical analysis

The normally distributed variables were expressed as mean ± standard deviation and continuous variables with skewed distribution as median with interquartile range (IQR). Categorical data were presented as frequency and proportions. Data analysis was done in a blinded manner by a statistician. Per-protocol and modified intention-to-treat (mITT) analyses were performed by last observation carried forward. Categorical variables between the two groups were compared by Fisher exact/Chi-square test(s). Normally distributed data were compared between groups using the Student’s *t*-test. The change in continuous variables (non-normal) within a group was evaluated by the Wilcoxon signed-rank test and between groups by the Mann–Whitney *U* test. A *P*-value of <0.05 was considered statistically significant. Data were analysed using STATA software (version 14, StataCorp LP, College Station, TX, USA) and IBM SPSS statistics software (version 21, Chicago, IL, USA).

## Results

**Figure 1.**
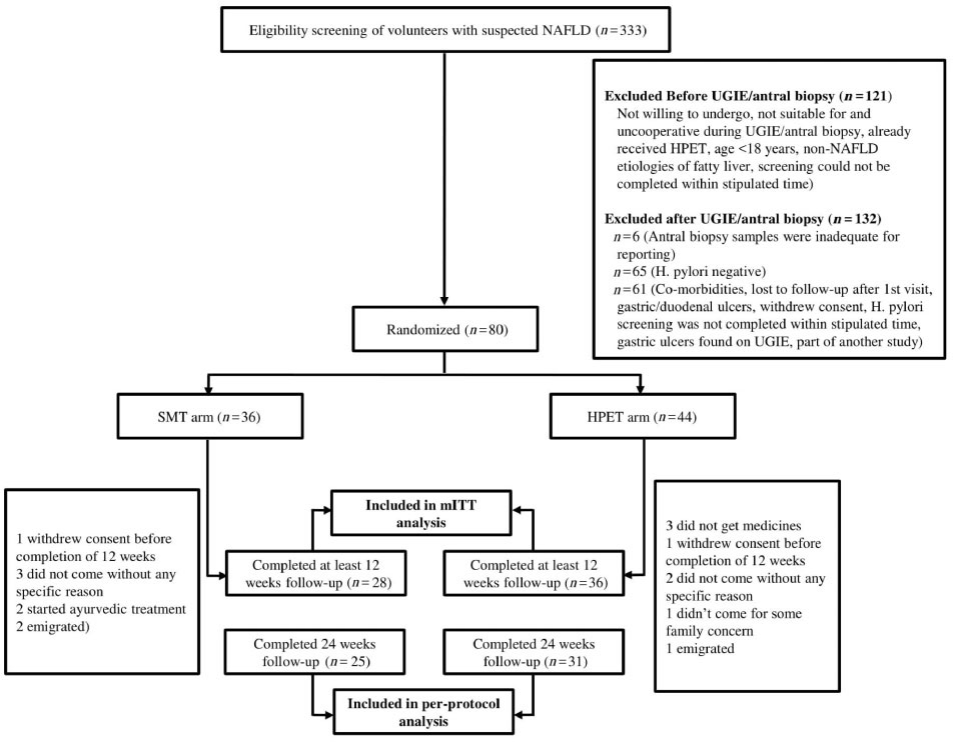
Consort chart showing the disposition of patients. UGIE, upper gastrointestinal endoscopy; NAFLD, non-alcoholic fatty liver disease; SMT, standard management therapy; HPET, *H. pylori*-eradication therapy; mITT, modified intention-to-treat.

A total of 333 suspected NAFLD patients, evaluated between June 2017 and July 2018, were screened for inclusion in the study. The last follow-up was completed in December 2018. Of these, 294 were diagnosed as NAFLD. Antral biopsy samples of 206 patients were tested for *H. pylori* infection, of whom 141 (68.4%) were found positive. Of these *H. pylori*-positive patients, finally, 80 NAFLD patients with *H. pylori* were randomized to either SMT (*n *=* *36) or SMT with HPET (*n *=* *44). The reasons for exclusion of other patients are shown in the CONSORT chart (Figure 1).

Sixty-four participants who received prescribed intervention and were followed at least until the second follow-up visit (i.e. 12 weeks) were included in the mITT analysis (36 in the HPET group and 28 in the SMT group). Twenty-four participants of 36 responded to HPET at 6 weeks, 11 did not respond, and, for one participant, eradication could not be assessed. Fifty-six participants who completed the study (followed up until 24 weeks) were included in the per-protocol analysis (31 in the HPET group and 25 in the SMT group). Overall, baseline clinical characteristics and anthropometric parameters of participants were similar in both study groups, except that a higher percentage of patients in the SMT group were obese and the waist/hip ratio was also higher as compared to the HPET group (Table 1).

**Table 1.** Baseline clinical characteristics of participants (modified intention-to-treat groups)

Characteristic	SMT group (*n* = 28)	HPET group (*n* = 36)	*P*-value
Age, years	42.2 ± 8.4	37.9 ± 9.5	0.066
Females	8 (28.6)	12 (33.3)	0.683
Aspartate aminotransferase, IU/L	32 (26–48)	37 (27–50)	0.481
Alanine aminotransferase, IU/L	45 (32–71)	53 (32–76)	0.390
Total cholesterol, mg/dL	181.4 ± 41.8	186.3 ± 30.8	0.598
Creatinine, mg/dL	0.8 ± 0.1	0.8 ± 0.2	0.417
Fasting blood sugar, mg/dL	97.4 ± 10.8	104.5 ± 8.6	0.208
HbA1c, %	5.6 ± 0.4	5.7 ± 0.6	0.480
Vitamin D, ng/mL	18.9 ± 16.4	19.6 ± 15.4	0.872
Body mass index, kg/m^2^	27.7 (26.3–30.4)	27.5 (24.6–30.7)	0.797
Waist/hip ratio	1.00 ± 0.07	0.96 ± 0.08	0.024
Basal metabolic rate, kcal	1,581.9 ± 177.8	1,635.8 ± 203.5	0.327
Fat, % of body weight	27.4 ± 7.3	25.1 ± 8.6	0.327
Fat mass, kg	20.3 ± 6.5	19.1 ± 9.3	0.602
Fat free mass, kg	53.4 ± 7.7	54.5 ± 8.2	0.633
Total body water, kg	39.1 ± 5.6	39.9 ± 5.9	0.640
Hypertension	0 (0.0)	4 (9.1)	0.125
Dyslipidemia^a^	25/27 (92.6)	33/34 (97.1)	0.579
Obesitya^b^	25 (89.3)	24 (66.7)	0.041

Values are expressed as mean ± standard deviation, median (interquartile range) or *n* (%).

^a^ Dyslipidemia was defined as total cholesterol >200 mg/dL, triglyceride >150 mg/dL, low-density lipoprotein >100 mg/dL, or high-density lipoprotein <40 mg/dL for males and <50 mg/dL for females.

^b^ Obesity was defined as body mass index ≥25 kg/m^2^.

SMT, standard management therapy; HPET, *H. pylori*-eradication therapy.

### Primary outcome

**Figure 2.**
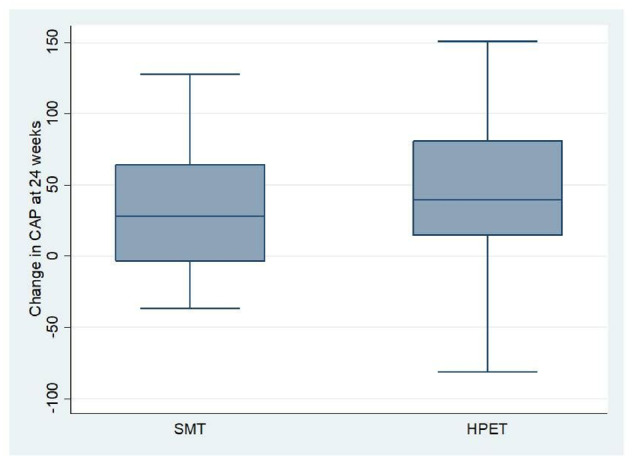
Box and Whisker plot showing a comparison of the median change in controlled attenuation parameter (CAP) value from the baseline to 24 weeks between two study groups. Change in CAP value (baseline–24 weeks) was not significantly different between the two study groups.

The median (IQR) CAP scores at baseline and 24-week CAP scores among patients in the SMT group were 329 (302–353) dB/m and 297 (278–328) dB/m, respectively, and in the HPET group were 326 (303–349) dB/m and 272 (249–319) dB/m, respectively. Both the SMT and the HPET groups had a significant reduction in CAP scores over the 24-week interval time (*P *=* *0.002 and *P* < 0.001, respectively, for the SMT and HPET groups). At 24 weeks, the median (IQR) decline in the CAP values was higher in the HPET group as compared to the SMT group (39.5 [13–82] dB/m vs 28 (–4–64) dB/m), but the difference in the change was not statistically significant (*P *=* *0.213; Table 2 and Figure 2). Similar results on CAP were noted on per-protocol analysis (Supplementary Table 1).

**Table 2.** Comparison of baseline, 24 weeks, and change in the parameters between the two study groups as per modified intention-to-treat analysis

Parameter	SMT group (*n* = 28)	HPET group (*n* = 36)	*P*-value
Controlled attenuation parameter, dB/m			
Baseline	329 (302–353)	326 (303–349)	0.771
24 weeks	297 (278–328)^a^	272 (249–319)^a^	0.124
Change	28 (–4–64)	39.5 (13–82)	0.213
Liver stiffness measurement, kPa			
Baseline	5.6 (4.7–7.6)	5.5 (4.8–6.5)	0.560
24 weeks	5.3 (4.7–6.3)	4.9 (4.2–6.2)^a^	0.368
Change	0.2 (–0.4–1.3)	0.9 (–0.7–1.9)	0.670
Body mass index, kg/m^2^			
Baseline	27.7 (26.3–30.4)	27.5 (24.6–30.7)	0.797
24 weeks	27.0 (24.2–29.8)	25.8 (23.1–29.9)^a^	0.357
Change	0.33 (–0.35–1.90)	0.98 (0.22–2.17)	0.096
Fat mass, kg			
Baseline	18.5 (15.3–23.4)	14.9 (12.6–22.6)	0.115
24 weeks	18.5 (13.6–25.4)	14.8 (11.7–28.6)	0.360
Change	0 (–0.7–1.7)	0.3 (–0.4–2.4)	0.582
HOMA-IR			
Baseline	1.6 (1.0–2.5)	1.9 (1.4–2.7)	0.222
24 weeks	1.8 (1.2–2.5)	1.6 (1.1–2.8)	0.934
Change	0 (–0.67–0.32)	0.12 (–0.02–0.53)	0.085
Aspartate aminotransferase, IU/L			
Baseline	32 (26–48)	37 (27–50)	0.481
24 weeks	28 (23–37)^a^	31 (23–38)^a^	0.570
Change	4.5 (0–12)	4 (0–21)	0.775
Alanine aminotransferase, IU/L			
Baseline	45 (32–71)	53 (32–76)	0.390
24 weeks	36 (20–52)^a^	42 (21–54)^a^	0.720
Change	3.5 (0–20)	7.5 (–2–38.0)	0.583
Total cholesterol, mg/dL			
Baseline	173 (155–216)	187 (166–210)	0.457
24 weeks	169 (148–198)	178 (163–199)	0.282
Change	0 (–7–20)	0 (–10–20)	0.878
Triglyceride, mg/dL			
Baseline	131 (89–211)	157 (113–204)	0.416
24 weeks	121 (94–154)	135 (112–185)	0.071
Change	1 (–14–57)	4.5 (–18–26)	0.558
Low-density lipoprotein, mg/dL			
Baseline	112 (93–139)	125 (111–144)	0.225
24 weeks	107 (88–130)	115 (102–135)	0.214
Change	1 (–1–9)	0 (–4–21)	0.948
High-density lipoprotein, mg/dL			
Baseline	40 (37–49)	41 (37–47)	0.966
24 weeks	41 (36–52)	43 (37–50)	0.743
Change	0 (–3.5–3.5)	0 (–3.7–1.0)	0.644
TNF-α, pg/mL			
Baseline	82.7 (46.3–174.1)	31.8 (19.4–84.5)	0.089
24 weeks	59.5 (16.3–171.0)	45.8 (23.9–70.1)	0.571
Change	0 (–15.7–6.4)	0 (–36.4–0)	0.733
Reduced glutathione, μg/mL			
Baseline	192.4 (151.4–213.1)	159.0 (143.2–183.1)	0.095
24 weeks	196.7 (181.0–213.7)	188.5 (167.4–221.3)*	0.570
Change	–16.4 (–56.1–1.9)	–38.4 (–114.5–-1.5)	0.364
Adiponectin, μg/mL			
Baseline	39.9 (17.6–51.5)	28.0 (17.8–45.4)	0.556
24 weeks	36.0 (27.6–58.2)	34.3 (24.4–45.3)	0.339
Change	–19.7 (–37.6–0)	–2.3 (–24.6–0)	0.228

All values are expressed as median (interquartile range). Change: baseline value–24 weeks’ value.

^a^ Comparison within group (24 weeks vs baseline), *P *<* *0.05.

SMT, standard management therapy; HPET, *H. pylori*-eradication therapy; HOMA-IR, Homeostatic Model Assessment-Insulin Resistance Index; TNF-α, tumor necrosis factor-alpha.

### Secondary outcomes

At 24 weeks, there was a significant reduction in BMI in the HPET groups as compared to the baseline value (*P *<* *0.001; Table 2); however, changes in the BMI over 24 weeks were not statistically different between the two groups (*P *=* *0.096). The liver enzymes were found to be reduced significantly at 24 weeks from baseline values in both the groups, but the changes between the groups were not different. There was no significant change in the lipid parameters within the groups and between the groups at 24 weeks. A significant increase in the serum levels of reduced glutathione was noted at 24 weeks in the HPET group but not in the SMT group, though, again, the changes between the two groups were not statistically different. Other parameters did not show significant improvement at 24 weeks from their baseline values in either of the study groups (Table 2). There was no difference in the results on per-protocol analysis (Supplementary Table 1).

### Subgroup analysis between HPET-treatment responders and combined non-responders plus SMT patients

Only 24 of 35 patients (68.6%) in the HPET group achieved *H. pylori* eradication (tested negative for *H. pylori* at 6 weeks) in the mITT population and none of the responders was found have re-infection at 12 and 24 weeks. Therefore, in order to assess the impact of *H. pylori* eradication, we compared the outcome parameters between HPET responders (*n *=* *24) and combined non-responders (*n *=* *11) plus the SMT group (*n *=* *28).

A significant improvement in HOMA-IR was noted in the HPET-responder group as compared to the combined groups (*P *=* *0.007; Table 3). *Helicobacter* *pylori* eradication resulted in improvement in the median HOMA-IR from 1.8 to 1.3 (*P *=* *0.046). The mean CAP score significantly improved in both the groups; however, change in the CAP score at 24 weeks was not significantly different between the two groups. Similarly, BMI and liver enzymes were reduced significantly at 24 weeks from baseline values in both the groups, but the changes were not significantly different between the groups. There was no significant change in the lipid parameters in both groups at 24 weeks (Table 3).

**Table 3.** Comparison of baseline, 24 weeks, and change in parameters between HPET-responder and non-responder plus SMT cases as per modified intention-to-treat analysis

Parameter	HPET-responder group (*n* = 24)	HPET-non- responder plus SMT group (*n* = 39)	*P*-value
Controlled attenuation parameter, dB/m			
Baseline	327 (303–345)	328 (300–354)	0.630
24 weeks	266 (249–316)^a^	297 (272–328)^a^	0.148
Change	38 (11–79)	29.5 (–1–65)	0.567
Liver stiffness measurement, kPa			
Baseline	5.5 (4.8–6.5)	5.6 (4.7–7.1)	0.650
24 weeks	4.7 (3.9–5.7)^a^	5.3 (4.7–6.3)	0.099
Change	1.2 (0.3–2.1)	0.2 (–0.5–1.3)	0.071
Body mass index, kg/m^2^			
Baseline	27.5 (24.8–30.7)	27.6 (25.3–30.2)	0.876
24 weeks	27.0 (23.7–29.9)^a^	27.7 (24.1–29.3)^a^	0.959
Change	0.9 (0–1.9)	0.7 (–0.2–2.1)	0.538
Fat mass, kg			
Baseline	15.7 (12.6–29.0)	18.3 (14.8–23.3)	0.592
24 weeks	16.3 (13.4–30.5)	17.8 (12.5–24.6)	0.700
Change	0.9 (0.3–2.7)	0 (–0.6–2.5)	0.456
HOMA-IR			
Baseline	1.8 (1.4–2.5)	1.6 (1.1–2.6)	0.412
24 weeks	1.3 (0.8–2.0)^a^	2.1 (1.2–2.5)	0.058
Change	0.6 (0.2–1.8)	–0.09 (–0.8–0.2)	0.007
Aspartate aminotransferase, IU/L			
Baseline	38 (26–50)	33 (27–50)	0.876
24 weeks	26 (23–38)^a^	30 (25–38)^a^	0.425
Change	6 (0–24)	4 (0–12)	0.291
Alanine aminotransferase, IU/L			
Baseline	47 (27–72)	46 (32–72)	0.708
24 weeks	26 (19–51)^a^	42 (21–54)^a^	0.205
Change	9 (–3–43)	5 (0–20)	0.535
Total cholesterol, mg/dL			
Baseline	186 (167–206)	176 (155–219)	0.834
24 weeks	176 (163–198)	171 (149–197)	0.435
Change	11.5 (–17–33)	0 (–7–17)	0.647
Triglyceride, mg/dL			
Baseline	133 (113–170)	151 (92–225)	0.718
24 weeks	141 (126–182)	122 (92–156)^a^	0.067
Change	12.5 (–30, 33)	11 (–9–57)	0.382
Low-density lipoprotein, mg/dL			
Baseline	122 (111–144)	116 (103–150)	0.511
24 weeks	113 (99–134)	109 (92–127)	0.508
Change	16.9 (–12–32)	1 (–3, 11)	0.449
High-density lipoprotein, mg/dL			
Baseline	41 (37–46)	42 (38–52)	0.337
24 weeks	44 (36–51)	42 (36–52)	0.974
Change	–1.5 (–5–1)	0 (–4–4)	0.480
TNF-α, pg/mL			
Baseline	31.7 (16.7–84.6)	60.4 (22.3–171.6)	0.208
24 weeks	39.7 (20.1–67.7)	49.3 (18.4–168.9)	0.198
Change	0 (–11.8–28.5)	0 (–62.6–25.7)	0.742
Reduced glutathione, μg/mL			
Baseline	182.0 (145.1–205.4)	163.5 (145.7–201.0)	0.733
24 weeks	187.0 (167.4–215.4)	198.5 (183.0–216.3)	0.415
Change	–6.6 (–39.3–8.7)	–42.4 (–83.7–1.1)	0.230
Adiponectin, μg/mL			
Baseline	41.8 (22.6–52.6)	28.1 (17.1–48.8)	0.560
24 weeks	32.2 (24.6–52.6)	36.6 (30.9–51.5)	0.288
Change	–2.3 (–25.7–0)	–20.7 (–35.4–0)	0.189

All values are expressed as median (interquartile range). Change: baseline value–24 weeks’ value.

^a^ Comparison within group (24 weeks vs baseline), *P *<* *0.05.

SMT, standard management therapy; HPET, *H. pylori-*eradication therapy; HOMA-IR, Homeostatic Model Assessment-Insulin Resistance Index; TNF-α, tumor necrosis factor-alpha.

### Compliance assessment

Compliance to both the dietary and exercise advice declined at the third follow-up visit, i.e. 24 weeks. In the HPET group, 26 of 32 (81%) participants in the mITT group were found to be compliant to *H. pylori*-eradication therapy. Compliance of four participants could not be assessed in modified intention-to-treat analysis as participants did not bring the remaining combi-pack strips of the drugs (Supplementary Table 2).

### Safety assessment

The most commonly reported adverse event following randomization was pain in the abdomen. No death/life-threatening adverse event apart from traumatic injury (unrelated to the intervention) was reported during the study period in either of the study groups (Supplementary Table 3).

## Discussion

Our study found that successful eradication of *H*. *pylori* led to greater improvement in IR at 24 weeks compared to patients in the non-treated (SMT) or failed-treated groups. There was a significant improvement in the hepatic steatosis at 24 weeks, as assessed by CAP, among both the SMT and HPET groups. However, the change in the CAP score was not significantly different between the two groups. Also, improvement in the various other clinical, laboratory, anthropometric, and metabolic parameters was not significantly different between the two groups.

IR has been shown to play a pivotal role in the pathogenesis and progression of NAFLD. *Helicobacter* *pylori* infection is also one of the proposed mechanisms for pathogenesis and/or progression of NAFLD [4, 12, 13]. A higher prevalence of NAFLD is seen in *H. pylori*-positive persons than those who are negative [4, 14]. *Helicobacter* *pylori* infection has been linked to IR as well [15, 16]. In this proof-of-concept study, patients with diabetes and other co-morbidities were excluded. Diabetes is associated with IR and approximately two-thirds of diabetics may have NAFLD [1]. In order to remove the additive effect of diabetes in the causation of NAFLD, we excluded these patients from the present trial.

Both the SMT and HPET groups were almost similar in characteristics at baseline. Overall improvement in various anthropometric parameters was seen at 24 weeks in both the study groups, though the comparison of change between the two groups was not statistically significant. Jamali *et al.* [17] in their study also did not find a significant difference in change in anthropometric parameters or liver-fat scores at 8 and 24 weeks from baseline between two groups of patients receiving either only standard treatment or additional quadruple therapy. However, only dyspectic NAFLD patients were selectively included in this study; therefore, the results cannot be generalized to all NAFLD patients. Polyzos *et al.* found that *H. pylori*-eradication therapy had no long-term effect on hepatic steatosis, but a trend towards improvement in the NAFLD fibrosis score, homocysteine, serum glutamic oxaloacetic transaminase, and erythrocyte sedimentation rate was observed [18].

In the present study, a significant reduction in hepatic steatosis occurred in the HPET and SMT groups, but the change was not significantly different between the two groups. A number of factors may affect the study outcome, including the dosage of intervention prescribed, compliance to prescribed intervention(s), and the size of the sample to detect significance. In the present study, HPET was effective for eradication of *H. pylori* in approximately 68% of patients and compliance to diet prescription was significantly low in the HPET group as compared to the SMT group at 24 weeks, both of which may affect the outcome. It may also be possible that the role of *H. pylori* in the pathogenesis of NAFLD is minor. Therefore, its eradication alone may not be sufficient to have had a significant impact on hepatic steatosis as compared to dietary and lifestyle modifications. Moreover, for a response (improvement) to HPET to be statistically significant as compared to standard treatment may require a larger sample size. In a pilot study evaluating the effect of *H. pylori*-eradication therapy, the hepatic-fat fraction was not found to improve significantly from baseline to 12 months and the change was not significantly different between two study groups of *H. pylori*-positive and -negative NASH patients [18]. *Helicobacter* *pylori* infection is a common problem in India. Overall, 70% of NAFLD patients had coexisting *H. pylori* infection, which is comparable to that reported in the general Indian population [12]. We found a sub-optimal response to currently used triple therapy with an eradication rate of only 68%, which warrants selection and the use of a more effective combination-therapy regimen depending upon the local antibiotic-sensitivity pattern. Our study did find that successful eradication of *H. pylori* resulted in greater improvement in HOMA-IR at 24 weeks compared to non-responder and non-HPET-treated patients. There was also a greater reduction in the mean CAP score within the treatment-responder group; however, the change was not significantly different from the non-responder/non-treated group, which may be potentially because of the small sample size. *Helicobacter* *pylori* has been proposed to adversely affect the IR through multiple mechanisms including a reduction in the adiponectin levels [4]. The present study showed an increase in serum adiponectin levels following *H. pylori* eradication, though the increase was not statistically significant and the comparison of change between the two groups was not significant as well. In our study cohort, the median HOMA-IR was on the lower side (<1.9). Several studies have found that NAFLD does occur in individuals with minimal or no IR. However, it is important to note that we had excluded diabetic NAFLD and HOMA-IR, which reflect mainly hepatic IR and may miss out peripheral IR.

Oxidative stress is one of the steps contributing to the pathogenesis of NAFLD. The GSH level did not increase significantly in the SMT group. *Helicobacter* *pylori* may have a direct or indirect role in substrate overload lipotoxicity increasing the oxidative stress [19] so its eradication may be expected to increase GSH levels and reduce triglyceride levels. Though serum GSH levels increased significantly in the HPET group, comparison of the increase in GSH levels between the two study groups was not statistically significant.

The present study has certain limitations. First, persons with diabetes mellitus were excluded from the study. Thus, these results cannot be extrapolated to diabetics. Second, dietary compliance was measured subjectively (only by history) without any objective tool. Third, because of the unavailability of the placebo, the design of the present study was open-label. Fourth, being a pilot study, because of a lower number of participants (in the analysis), it may not have adequate power to detect a significant difference in study outcomes between the two study groups; studies with a larger sample size may confirm the findings. In this pilot study, we planned to include 100 patients in the proposed time frame and could recruit only 80, which was the reason for the unequal number of patients in the two arms. Recruitment was stopped after July 2018, in order to complete the study by December 2018. Fifth, we did not use the MRI-proton density fat fraction or liver biopsy for hepatic steatosis assessment, which has been reported to be better than CAP measurements. The median BMI was similar between the two groups (27.7 vs 27.5 kg/m^2^), whereas, when patients were classified as obese based on the BMI cut-off of 25, a higher percentage of patients in the SMT groups were obese as compared to those in the HPET group. It is possible that this difference could have affected the results in our study.

In conclusion, our randomized–controlled study revealed that *H. pylori*-eradication therapy was comparable to diet and exercise alone in terms of reducing hepatic steatosis and other metabolic parameters at 24 weeks in NAFLD patients. However, successful eradication of *H. pylori* could be achieved only in 68% of cases and, in those patients, IR improved significantly in a greater proportion. Additional work involving a larger sample size, more effective HPET regimen, and paired liver biopsy is needed for further understanding.

## Authors’ contributions

Study concept and design: V.M., P.G., R.K., Shalimar. Acquisition of data: V.M., P.G., B.N. Analysis and interpretation of data: Shalimar. Histological analysis of samples: P.D., R.Y. Statistical analysis: A.D.U. Drafting of manuscript: V.M., P.G., V.L.K., P.D., R.Y., B.N., R.K. Critical revision for important intellectual content: R.K., Shalimar. All authors read and approved the final manuscript.

## Funding

AIIMS Intramural grant [No. F.8-539/A-539/2017/RS].

## Supplementary Material

goz058_Supplementary_Data
