# Supplementary material for: Effect of Helicobacter pylori-eradication therapy on hepatic steatosis in patients with non-alcoholic fatty liver disease: a randomized–controlled pilot study
Source: Gastroenterol Rep (Oxf). 2019 Nov 25;8(2):104–10. doi: 10.1093/gastro/goz058 (PMC7261206; doi:10.1093/gastro/goz058)
Supplement: goz058_Supplementary_Data [file goz058_supplementary_data.docx]

**Supplementary Table 1**. Comparison of change in the parameters between two study groups at 24 weeks as per-protocol analysis.

| Parameter | SMT group (*n* = 25) | HPET group (*n* = 31) | *P*-value |
| --- | --- | --- | --- |
| ∆CAP, dB/m | 32 (-3–64) | 36 (11–83) | 0.373 |
| ∆LSM, kPa | 0.2 (0.4–1.2) | 1.0 (-0.8–2.0) | 0.473 |
| ∆Body mass index, kg/m^2^ | 0.48 (-0.6–2.1) | 0.88 (0.19–2.20) | 0.135 |
| ∆Fat mass, kg | 0 (-1.2–2.6) | 0.45 (-0.47–2.47) | 0.631 |
| ∆HOMA-IR | 0 (-0.66–0.27) | 0.16 (-0.34–0.63) | 0.091 |
| ∆AST, IU/L | 5 (0–13.5) | 4 (0–24) | 0.685 |
| ∆ALT, IU/L | 4 (0–20) | 10 (-3–43) | 0.541 |
| ∆Total cholesterol, mg/dL | 0 (-7.5–22.3) | 0 (-11–21) | 0.911 |
| ∆Triglyceride, mg/dL | 4 (-10.5–57.8) | 11 (-20–34) | 0.621 |
| ∆LDL, mg/dL | 2 (-1–14) | 0 (-4.0–21) | 0.847 |
| ∆HDL, mg/dL | 0 (-3–5) | 0 (-3–1) | 0.653 |
| ∆TNF-α, pg/mL | 0 (-47–19.2) | 0 (-42–69.7) | 0.971 |
| ∆GSH, μg/mL | -16.4 (-56.1–1.9) | -38.4(-114–-1.5) | 0.130 |
| ∆Adiponectin, μg/mL | -4.7 (-22.6–5.9) | 0 (-5.1–0) | 0.479 |

Note: all values are expressed as median (interquartile range). ∆: baseline value - 24 weeks value.

Abbreviations: SMT, standard management therapy; HPET, *H. pylori* eradication therapy; CAP, controlled attenuation parameter; LSM, liver stiffness measurement; HOMA-IR, homeostatic model assessment-insulin resistance index; AST, aspartate aminotransferase; ALT, alanine aminotransferase; LDL, low density lipoprotein; HDL, high density lipoprotein; TNF-α, tumor necrosis factor-alpha; GSH, reduced glutathione

**Supplementary Table 2**. Assessment of compliance.

| Characteristics | SMT arm (*n* = 28) | HPET arm (*n* = 36) | *P*-value |
| --- | --- | --- | --- |
| ***Diet and exercise compliance at 12 weeks*** | | | |
| Diet, compliant/total (%) | 22/27 (81.5%) | 26/36 (72.2%) | 0.393 |
| Exercise, compliant/total (%) | 21/27 (77.8%) | 27/36 (75.0%) | 0.798 |
| Exercise METs, median (min, max) | 890.3 (0, 7116) | 977.3 (0, 2878) | 0.753 |
| ***Diet and exercise compliance at 24 weeks*** | | | |
| Diet, compliant/total (%) | 17/27 (62.9%) | 13/36 (36.1%) | 0.035 |
| Exercise, compliant/total (%) | 16/26 (61.5%) | 25/36 (69.4%) | 0.516 |
| Exercise METs, median (min, max) | 816.7 (0, 4127.7) | 967.7 (0, 2911.7) | 0.648 |
| Compliance to HPET, compliant/total (%) | NA | 26/32 (81.2%) | - |

Abbreviations: SMT, standard management therapy; HPET, *H. pylori* eradication therapy; METs, metabolic equivalents of task; NA: not applicable

**Supplementary Table 3**. Adverse events reported/experienced by participants.

| Events | SMT arm (*n* = 28) | HPET arm (*n* = 36) |
| --- | --- | --- |
| Pain abdomen* | 4 (14.3%) | 7 (19.4%) |
| Altered bowel habits^#^ | 2 (7.1%) | 6 (16.7%) |
| Alteration of taste | 0 | 5 (13.9%) |
| Darkening of tongue, gums and face | 0 | 2 (5.6%) |
| Oral ulcerations/sores | 0 | 3 (8.3%) |
| Weakness/fatigue | 0 | 3 (8.3%) |
| Pain, swelling and/or paraesthesia in hands and feet | 1 (3.6%) | 4 (11.1%) |
| Headache/lightheadedness | 0 | 1 (2.7%) |
| Malaise | 0 | 5 (13.9%) |
| Decreased hearing and itching (both ears) and vertigo | 0 | 1 (2.8%) |
| Traumatic injury | 3 (10.7%) | 2 (5.6%) |

* Including flank pain and abdominal distension/bloating.

^#^ Including loose stools, increased frequency of stools, decreased appetite, sense of incomplete evacuation, excessive gas formation in abdomen.
